# Ultra-Processed Foods as a Cross-Cutting Driver of Health Inequities: Low- and Middle-Income Countries Across the Life-Course

**DOI:** 10.3390/nu18193235

**Published:** 2026-09-30

**Authors:** Lei Qian, Zimeng Cheng, Shiling Huang, Kailin Zhong, Bin Xue, Ankita Kumari, Abdul Rahaman

**Affiliations:** 1 School of Food & Pharmaceutical Science and Technology, Guangzhou College of Technology and Business, Guangzhou 510850, China; 2 College of Food Science, South China Agricultural University, Guangzhou 510642, Chinaxuebin@stu.scau.edu.cn (B.X.); 3 School of Food Science and Engineering, Foshan University, Foshan 528225, China; 4 Guangdong Key Laboratory of Food Intelligent Manufacturing, Foshan University, Foshan 528225, China; 5 School of Food Science and Engineering, South China University of Technology, Guangzhou 510641, China

**Keywords:** ultra-processed foods, human health, low- and middle-income countries (LMICs), nutrition, food policy

## Abstract

Ultra-processed foods (UPFs) have become a growing part of everyday diets worldwide and have been linked to obesity, non-communicable diseases, and adverse mental health outcomes. Yet their role in deepening nutritional and health inequalities across socioeconomic groups and different stages of life is still not fully addressed in policy discussions. Drawing on recent population-based studies, longitudinal surveys, and mechanistic evidence, this perspective examines how UPF consumption intersects with food insecurity, psychosocial distress, and neurodevelopmental health from pregnancy through adulthood. Evidence from the United States shows that adults experiencing food insecurity obtain a larger share of their daily energy from UPFs. At the same time, surveys from low- and middle-income countries (LMICs) show that many infants and young children are already consuming sugar-sweetened beverages. Other observational studies have linked higher UPF intake with addictive-like eating behaviours and symptoms of depression, anxiety, and stress, although these studies cannot yet prove causality. In many LMICs, ongoing dietary transitions are also contributing to a rapid increase in overweight and obesity among socioeconomically disadvantaged groups, even while childhood stunting declines only slowly. Together, these findings suggest that UPF exposure is not only a dietary issue, but also a marker of wider social and nutritional vulnerability across the life-course. Breaking this cycle will require coordinated policy action, including stronger restrictions on unhealthy food marketing, front-of-package warning labels, better regulation of foods marketed to infants and young children, reforms to food-assistance programmes, and fiscal measures that make minimally processed foods more affordable and accessible.

## 1. Introduction

Rapid urbanization, industrial food production, changing lifestyles, and the widespread availability of ready-to-eat products have profoundly transformed dietary habits worldwide. In high-income countries, UPFs have become a routine part of everyday eating. These products are often made from cheap, energy-dense ingredients and contain little, if any, whole or natural food. Among certain population groups, these products account for more than half of the total daily energy intake [1,2]. This trend is now rapidly spreading to LMICs as well, where sales of sugary drinks and other non-essential processed foods have surged over the past two decades [3]. This shift represents far more than a mere change in dietary habits; it reflects a profound transformation in food systems that is reshaping the relationship between food, health, and society.

According to the NOVA classification, ultra-processed foods are products made mostly, or sometimes entirely, from substances extracted from foods, chemically modified ingredients, and industrial formulations. They often contain additives that improve taste, texture, appearance, and shelf-life [4]. Growing evidence suggests that high UPF intake may be associated not only with physical health problems, but also with mental health concerns, addictive-like eating patterns, pleasure-driven eating, and possible effects on neurodevelopment, especially when these foods become a regular part of the diet from childhood [5,6,7]. At the same time, reliance on UPFs is closely tied to socioeconomic inequality. People do not consume these foods in the same way or for the same reasons; their choices are shaped by income, food availability, and the food environment around them. In the United States, for example, people experiencing food insecurity and those enrolled in the Supplemental Nutrition Assistance Program (SNAP) obtain a larger share of their daily calories from UPFs than food-secure individuals or those not eligible for income-based assistance [1]. Similarly, in many LMICs, infants and young children, who should ideally be introduced to unsweetened and minimally processed foods, are increasingly exposed to sugary drinks and ultra-processed snacks. This matters because eating patterns formed early in life can influence taste preferences, food choices, and the risk of metabolic diseases later in adulthood [3]. Recent comparative risk assessment data from 45 countries show that UPF consumption is increasing worldwide, with some of the fastest growth occurring in low- and middle-income countries. In these settings, ultra-processed foods are gradually replacing traditional and local diets. This shift should not be seen simply as a matter of personal choice; it reflects wider changes in food systems, including rapid urbanization, global food markets, and the growing influence of multinational food companies in emerging economies [8,9].

Evidence from the South Asia Biobank shows that ultra-processed foods are quickly becoming part of everyday diets across the region. In a study of 60,714 adults from Bangladesh, Pakistan, Sri Lanka, North India, and South India, about three out of four people in Bangladesh, Sri Lanka, and North India reported eating at least one UPF in the previous 24 h. Consumption was lower in South India and Pakistan, but still substantial, affecting around 40% of adults. Among people who consumed UPFs, these foods provided about 13–17% of their total daily energy intake, with the highest contribution seen in Pakistan. Biscuits were one of the most commonly consumed UPFs across all regions, while other major sources differed by location: sugar-sweetened beverages were more prominent in Pakistan, packaged savory snacks in South India, and breakfast cereals in Bangladesh [10]. These findings indicate that UPFs are no longer occasional foods in South Asia. Instead, they are becoming a routine part of daily diets in a region traditionally characterized by diverse, home-prepared, and minimally processed foods.

The rapid rise in UPFs in LMICs is especially worrying because it is happening alongside long-standing nutrition problems. Many of these countries now face a “double burden” of malnutrition: undernutrition and childhood stunting remain unresolved, while overweight, obesity, and diet-related non-communicable diseases are increasing quickly. The economic impact is also serious. By 2030, diet-related non-communicable diseases are expected to cost LMICs more than $4.8 trillion through lost productivity and higher healthcare spending. This added pressure could further weaken health systems that are already under strain. The rapid spread of UPFs into everyday food environments has become one of the major public health challenges of our time. Tackling this problem requires more than asking people to make “better choices.” It calls for coordinated structural action, including restrictions on unhealthy food marketing, clear front-of-pack nutrition labels, taxes on sugar-sweetened beverages, and stronger investment in sustainable local food systems. In this article, we examine how UPFs contribute to health inequalities across different stages of life.

This perspective brings together evidence from peer-reviewed studies published mainly between 2015 and 2025, identified through targeted searches in PubMed, Scopus, and Web of Science. We gave priority to population-based studies, longitudinal surveys, and mechanistic reviews that examine how UPF consumption intersects with health inequities and life-course health outcomes. Because this is a perspective rather than a systematic review, our aim is not to provide exhaustive coverage of all available evidence. Instead, we use the literature to highlight key patterns, identify important gaps, and propose a conceptual framework for future research.

## 2. The Unequal Distribution of UPF Consumption Across High-Income and LMICs

### The Nutrition Transition in LMICs

**Figure 1:**
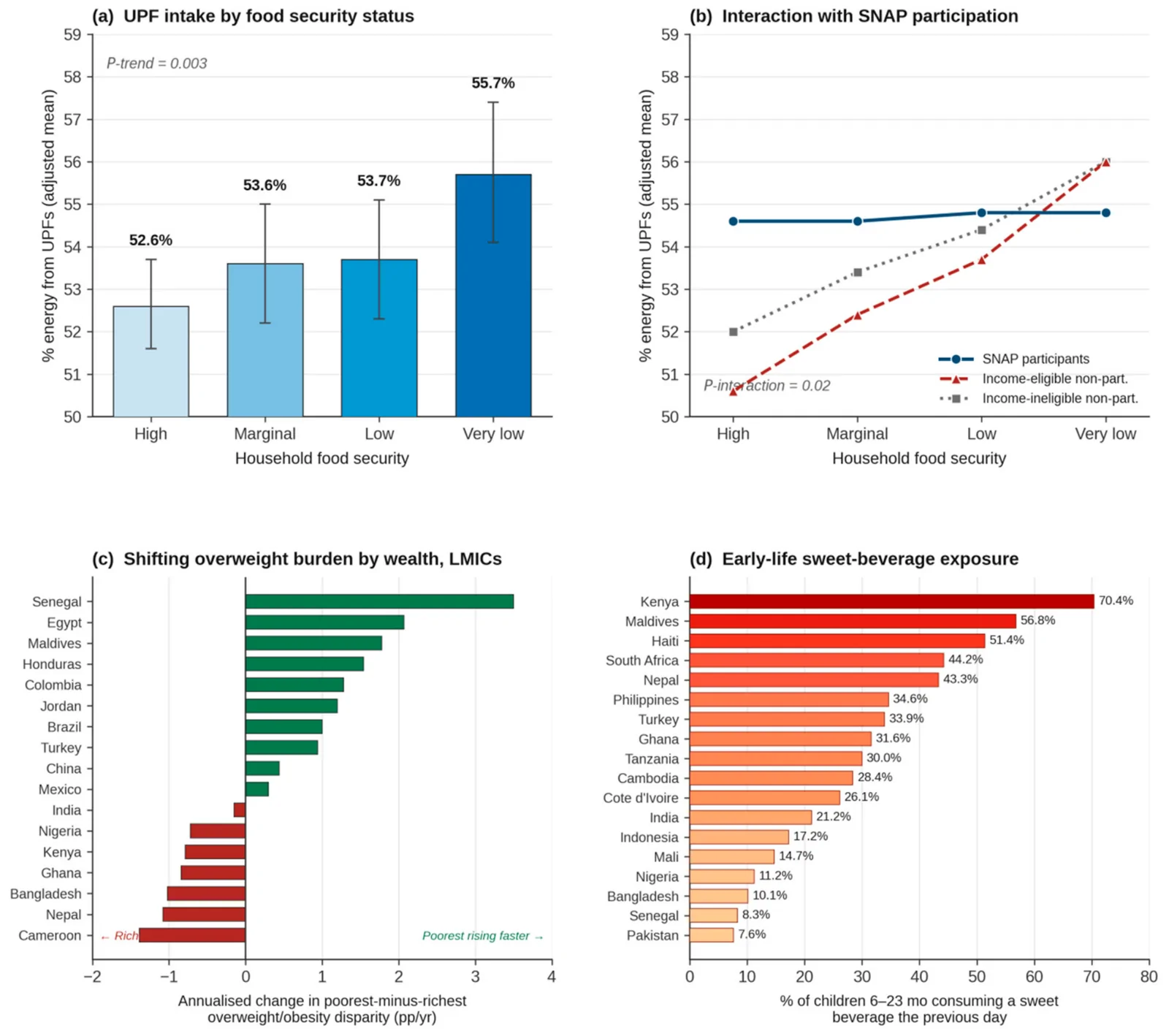
The unequal distribution of UPF consumption across the life-course. UPF intake by food security status and SNAP participation. Data from NHANES 2007–2016 (n = 9190). (**a**) Adjusted mean percentage of energy from UPFs across four levels of household food security, showing a dose–response increase. (**b**) Interaction plot: among income-eligible non-participants and income-ineligible non-participants, worsening food security is associated with higher UPF consumption; among SNAP participants, the gradient is flattened, and overall intake is high. Source: Leung et al. [1]. (**c**) Shifting burden of overweight/obesity by wealth in LMICs. Bars show the annualised change in the gap between the poorest and richest wealth quintiles in overweight/obesity prevalence among women aged 15–49 years. Positive (green) values indicate that overweight/obesity is rising faster among the poorest quintile, the emerging pattern in much of Latin America, East Asia, and the Middle East; negative (red) values indicate the richest quintile is still gaining faster, as remains the case across most of South Asia and sub-Saharan Africa. Source: Popkin and Laar [3]. (**d**) Early exposure to UPFs: percentage of children aged 6–23 months who consumed a sweet beverage the day before the survey, for a representative spread of LMICs from the most recent available DHS; the Ghana value (31.6%, navy outline) is from the 2022 Ghana DHS. Source: Popkin and Laar [3].

Dietary and nutritional patterns are changing rapidly in many low- and middle-income countries, a process often described as the “nutrition transition.” This shift has created a complicated public health challenge. Analysis of Demographic and Health Survey (DHS) data from 70 countries show that childhood stunting, measured as low height-for-age, is slowly declining, while overweight and obesity among women and children are rising quickly [3]. Importantly, this change is also reshaping the social pattern of malnutrition. In many Asian, Latin American, and Middle Eastern countries, overweight and obesity are now increasing faster among the lowest-income groups than among the wealthiest groups. This suggests that vulnerable communities are becoming more exposed to food environments dominated by cheap, convenient, and ultra-processed foods, which may be contributing to rising obesity and diet-related disease risk (Figure 1c).

The consumption of UPFs among preschool-aged children is also rising quickly, raising serious concerns about the formation of unhealthy eating habits early in life. National survey data show that many children aged 6–23 months are already consuming sugary drinks (Figure 1d). For example, the 2022 Ghana DHS reported that 31.6% of infants and young children had consumed a sugary drink on the previous day [11]. This shift is taking place alongside the rapid commercial expansion of the infant and young child food market. At the same time, added sugars are becoming increasingly common in commercial foods marketed for infants and young children, particularly in LMICs [3]. Many of these products are heavily processed and are marketed as appropriate for young children, even though their added sugar and sodium levels often exceed WHO recommendations [12]. This early exposure is concerning because it can normalize very sweet or salty tastes during a period when food preferences are still being formed. Similar inequalities are also visible in adult diets. In an analysis of 9190 US adults from NHANES 2007-2016, ultra-processed foods contributed 52.6% of total energy intake among people with high food security and 55.7% among those with very low food security after adjustment for covariates (P-trend = 0.003). The largest differences were observed for pizza and soft drinks (Figure 1a,b) [1]. Among SNAP participants, UPFs provided 54.7% of total energy, compared with 53.0% among income-eligible non-participants. SNAP participation also modified the association between food insecurity and UPF intake (P-interaction = 0.02): UPF intake increased with food insecurity among non-participants, but this pattern was not observed among SNAP participants [1]. These findings suggest that SNAP may buffer the dietary effects of worsening food insecurity, although UPF consumption remains high, highlighting the need for food-assistance programmes to improve access to affordable, minimally processed foods.

## 3. NOVA Classification: Utility, Debate and Relevance to Health Inequities

Food processing is not inherently harmful. Many processing technologies, including pasteurization, drying, freezing, fermentation, packaging, and fortification, have improved food safety, shelf-life, convenience, nutrient delivery, and food security. Therefore, the public health concern is not food processing as a broad technological practice, but the increasing displacement of minimally processed foods and freshly prepared meals by industrially formulated ultra-processed foods.

The NOVA classification was developed to categorize foods according to the nature, extent, and purpose of industrial processing. It distinguishes unprocessed or minimally processed foods, processed culinary ingredients, processed foods, and ultra-processed foods. Within this framework, ultra-processed foods are generally defined as industrial formulations made from refined food substances, modified ingredients, and cosmetic additives, often designed to be convenient, inexpensive, highly palatable, shelf-stable, and strongly marketed [4,13].

At the same time, NOVA remains debated within food science and nutrition. Critics argue that the classification may not always separate processing intensity, ingredient number, food formulation, and nutritional quality with sufficient precision. Some processed foods may contribute positively to dietary quality, particularly when fortified or reformulated, while some home-prepared foods may still be high in sugar, salt, saturated fat, or energy density. Thus, NOVA should not be interpreted as a complete substitute for nutrient profiling, dietary-pattern assessment, or food-safety evaluation [14].

Despite these limitations, NOVA remains useful for the present perspective because it captures a broader food-system problem that nutrient-based approaches alone may miss. Ultra-processed foods are not only defined by their nutrient composition, but also by their industrial formulation, affordability, convenience, marketing, and capacity to displace traditional dietary patterns. These features are especially relevant in low- and middle-income countries, where rapid urbanization, expanding retail markets, aggressive food marketing, and socioeconomic vulnerability are reshaping food environments across the life-course. Accordingly, this article uses NOVA cautiously as a public-health framework to examine how UPF exposure may contribute to diet-related health inequalities, while recognizing that the health effects of individual foods depend on their composition, context, and patterns of consumption.

## 4. Psychosocial Correlates of UPF Consumption

**Figure 2:**
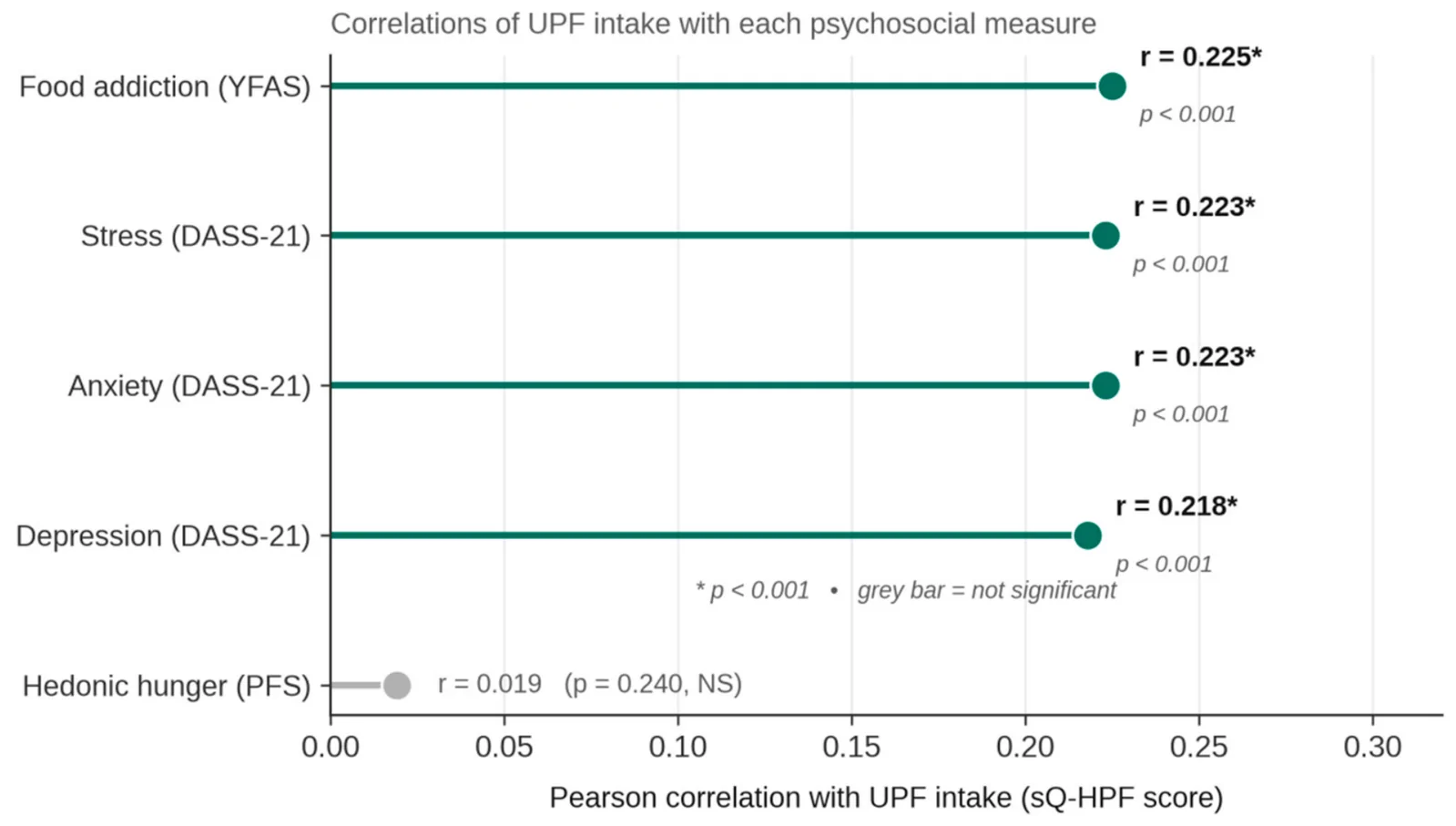
Correlations between UPF consumption and psychosocial factors. Pearson correlations of UPF intake (sQ-HPF score) with each psychosocial measure, from a cross-sectional study of 3997 Turkish adults (mean age 31.7 years). UPF intake is significantly positively correlated with food addiction (YFAS; r = 0.225), stress (r = 0.223), anxiety (r = 0.223), and depression (r = 0.218), all *p* < 0.001, but shows no significant correlation with hedonic hunger (PFS; r = 0.019, *p* = 0.24). Source: Mengi Çelik et al. [7].

Price, convenience or availability alone cannot explain the high consumption of UPFs among vulnerable groups. A cross-sectional study involving 3997 Turkish adults (mean age 31.7 years) found that UPF consumption measured using the ‘Screening Questionnaire of Highly Processed Food Consumption’ was positively associated with food addiction scores on the ‘Yale Food Addiction Scale’ (r = 0.225, *p* < 0.001) and with subscale scores for depression, anxiety, and stress (r = 0.218–0.223; all *p* < 0.001) (Figure 2). Individuals with higher UPF consumption were predominantly women, younger adults, those living alone, and the unemployed. In multivariable linear regression analysis, gender, age, food addiction, and depression, anxiety, and stress scores emerged as independent predictors of UPF consumption; conversely, hedonic hunger (measured by the ‘Power of Food Scale’) showed no significant association with UPF intake (r = 0.019, *p* = 0.24) and did not serve as a predictor [7]. This pattern suggests that, in this population, the drive for UPFs may be linked more to addiction-like processes and emotional regulation than to the anticipated reward derived from eating. However, due to the cross-sectional nature of the study, causal inferences cannot be drawn.

These findings align with the view held by Leung and others that food insecurity acts as a form of chronic stress. It activates the hypothalamic–pituitary–adrenal axis and promotes “comfort eating” that is, the consumption of highly palatable, energy-dense foods [1]. The combination of stress, food addiction, and an environment saturated with UPFs creates a vicious cycle: psychological distress increases UPF consumption, which can further deteriorate mental health and reinforce addictive eating habits [15].

### Impact on Brain Development Across the Life-Course

The evidence linking UPF consumption with adverse health outcomes comes from different study types and should be interpreted accordingly. Human observational evidence is substantial. Dai et al. updated 39 meta-analyses using 122 articles across 49 health outcomes and reported convincing evidence for associations with renal function decline (OR: 1.25; 95% CI: 1.18–1.33) and wheezing in children/adolescents (OR: 1.42; 95% CI: 1.34–1.49), with highly suggestive evidence for diabetes, overweight, obesity, depression, and common mental disorders [16]. Similarly, Lane et al. reviewed 45 pooled analyses from 14 meta-analyses involving approximately 9.9 million participants and found higher UPF exposure associated with 32 adverse outcomes, including cardiovascular mortality, type 2 diabetes, obesity and mental health disorders [17].

Experimental human evidence is more limited but important. In a controlled inpatient crossover trial of 20 adults, Hall et al. found that an ultra-processed diet increased ad libitum energy intake by 508 +/− 106 kcal/day (*p* = 0.0001) and produced weight gain of 0.9 +/− 0.3 kg over two weeks, whereas the unprocessed diet led to comparable weight loss [18]. This supports a short-term causal effect on energy intake and body weight, but not necessarily on all long-term outcomes.

Mottis et al. [5] stated that maternal UPF intake during pregnancy is associated with adverse neurodevelopmental outcomes in offspring, including deficits in overall cognitive function, verbal expression, and memory during the preschool years. Regarding mechanisms, UPFs can disrupt fetal brain development through several pathways such as placental inflammation, oxidative stress, epigenetic modifications, and alterations in the gut microbiome, mechanisms that have primarily been demonstrated in animal models [5,19]. Key nutrients essential for synaptic transmission such as long-chain polyunsaturated fatty acids, choline, iron, and zinc are often displaced by UPF-rich diets, and deficiencies in these nutrients during the critical third-trimester period can lead to irreversible cognitive deficits [20].

During adolescence, a second critical period, dietary patterns rich in UPFs are associated with deficits in executive functions and increased symptoms of hyperactivity and inattention [21]. The adolescent brain, characterized by heightened sensitivity to reward and a prefrontal cortex that is still maturing, may be particularly vulnerable to the hyper-addictive striatal reward response triggered by the fat-sugar combinations found in UPFs (an effect observed in adults) [22]. This can accelerate the shift from goal-directed to habitual eating, thereby establishing the neural basis for lifelong dietary preferences and obesity.

In adulthood, sustained consumption of Western-style diets rich in UPFs is linked to structural brain changes, including reduced hippocampal volume, and high UPF intake is associated with an increased risk of dementia [23,24]. The gut–brain axis plays a key mediating role: UPF-induced dysbiosis increases intestinal permeability and systemic inflammation, which in turn promotes neuro-inflammation and neurodegeneration [5,25]. Animal and mechanistic studies further suggest plausible pathways involving insulin resistance, hepatic steatosis, gut-microbiome disruption, inflammation, oxidative stress, and gut–brain axis signaling. However, many animal models use cafeteria-style, high-fat, high-sugar, or high-sodium diets, so they cannot fully isolate ultra-processing itself. Evidence from Western dietary patterns, sugar-sweetened beverages, sodium, saturated fat, low fiber intake, and food additives is relevant but should be treated as supportive rather than direct UPF evidence. Therefore, throughout this perspective, we distinguish direct human UPF evidence from animal findings, related dietary exposures, and plausible mechanisms.

## 5. Policy Levers to Break the Cycle

Addressing UPF-driven health inequities requires a multi-sectoral, life-course-oriented policy agenda. We highlight four interconnected strategies:

### 5.1. Protect the Youngest: Mandatory Standards for Infant and Toddler Foods

Global sales in the infant and toddler feeding sector increased from US$33.2 billion in 2010 to US$67.9 billion in 2022, while the global baby food market specifically grew from US$9.6 billion to US$17.9 billion over the same period. Emerging economies now account for approximately half of global baby food sales, and more than 90% of growing-up formula sales in 2022 occurred in low- and middle-income countries. These trends indicate a rapid commercialization of infant and toddler feeding, including the growing use of infant formula, follow-on or growing-up formula, and packaged complementary foods. Many commercial infant and toddler foods vary in nutritional quality, but concerns have been raised about added sugars, sweet taste profiles, additives, and ultra-processed ingredients in some products. Stronger composition standards, clearer labelling, and restrictions on inappropriate marketing are therefore needed to reduce early exposure to unhealthy dietary patterns while supporting breastfeeding and appropriate complementary feeding [26]. A recent US survey revealed that nearly half of the energy intake from complementary foods for children aged 6 to 23 months comes from ultra-processed products. Although these provide a significant portion of UPFs’ iron and zinc, particularly through fortified breakfast cereals. These often contain high amounts of sugar, sodium, and refined grains; in contrast, fruits, vegetables, and dairy products are mostly from unprocessed or minimally processed sources. With the commercial baby-food sector growing rapidly, these findings reinforce the need for standards that limit added sugar, sodium, and other unnecessary additives, while promoting nutrient-rich, less processed options for the youngest consumers [27]. The WHO recommends that children aged 0 to 24 months should avoid added sugars; however, UPFs marketed for young children and infants often contain excessive sugar and sodium [12,28]. The scope of the International Code for Marketing of Breast-milk Substitutes should be expanded to cover all commercial food and drink products for children under 36 months of age, and a front-of-pack warning label should be required for any product that exceeds the age-recommended limits for added sugars or sodium. The WHO Nutrient and Promotion Profile Model (NPPM) provides a ready-made framework for developing and implementing nutritional standards for such products [3,29].

### 5.2. Reform Food Assistance Programs to Prioritize Nutrition

The global rise in UPFs has displaced traditional dietary patterns based on fresh and minimally processed foods, contributing to poorer diet quality and increased health risks. While current policies mainly target nutrients such as sugar, sodium, and unhealthy fats, a broader food-system approach is needed to address the production, marketing, and availability of UPFs. Effective strategies should combine product regulation, healthier food environments, corporate accountability, and support for affordable fresh foods, particularly for vulnerable populations, with interventions tailored to national food-system contexts [30]. Food assistance programmes in low- and middle-income countries should move beyond calorie provision and be redesigned to improve dietary quality while reducing dependence on UPFs. Evidence from food assistance and nutrition-sensitive social protection shows that cash transfers, vouchers, school meals, and in-kind support can improve food access, but their nutritional effects depend on transfer adequacy, food quality, targeting, and complementary actions such as nutrition education and local food-system support [31,32]. Because UPF markets are expanding rapidly in many LMICs and are displacing diets based on fresh and minimally processed foods, food assistance should include nutrition standards, healthier procurement, and incentives for fruits, vegetables, pulses, eggs, milk, and other minimally processed foods [33]. Such reforms should be gradual and context-specific, especially where affordable alternatives are limited, and should be paired with policies that improve the availability, affordability, and convenience of minimally processed foods rather than placing responsibility only on vulnerable households [34].

### 5.3. Implement Fiscal and Regulatory Measures to Reshape Food Environments

To improve the food environment, economic and legal measures are needed to curb the dominance of UPFs and promote healthy eating habits. UPFs typically contain high amounts of added sugar, sodium, unhealthy fats, and energy density, while being deficient in fiber and essential nutrients; these are strongly linked to an increased risk of obesity and non-communicable diseases (NCDs). Although fortification has increased micronutrient levels in UPFs in regions like South Africa, it has failed to address the major nutritional and environmental concerns associated with them. Policy has primarily focused on taxes; while over 45 countries have taxed sugar-sweetened beverages, very few have targeted other categories of UPFs. Effective strategies should combine economic measures with front-of-pack warning labels, marketing restrictions, healthy school food policies, and subsidies to facilitate access to affordable, fresh, and less processed foods, especially for vulnerable populations [35]. The rising global prevalence of UPFs has displaced traditional dietary patterns based on fresh and minimally processed foods, leading to a decline in diet quality and increased health risks. Current policies primarily target nutrients such as sugar, sodium, and unhealthy fats; however, a comprehensive food-system approach is required to address the production, marketing, and availability of UPFs. Effective strategies should encompass product regulation, healthy food environments, corporate accountability, and support for affordable fresh foods, particularly for vulnerable populations, alongside interventions tailored to national food-system contexts [30,33].

A combined package of fiscal and regulatory measures is likely to be more effective than any single intervention. Taxes on sugar-sweetened beverages and discretionary UPFs, when paired with subsidies for fruits, vegetables, pulses, and other minimally processed foods, can shift relative prices in ways that encourage healthier purchasing patterns and improve diet-related outcomes [36]. These measures should be reinforced by front-of-package warning labels, restrictions on child-directed marketing, and limits on the sale or promotion of unhealthy products in schools, all of which have gained policy momentum in Latin America and other regions [37]. Experiences from Chile, Mexico, and Brazil further show that sustained political commitment, clear regulatory design, and civil society advocacy are critical for advancing nutrition policies despite strong industry opposition.

### 5.4. Integrate Mental Health and Nutrition Services

UPF consumption should be addressed not only as a metabolic risk factor but also as a potential contributor to poor mental health. Recent reviews show that higher UPF intake is associated with increased odds of depressive and anxiety symptoms, although causal pathways remain under investigation [38]. Proposed mechanisms include poor nutrient density, glycaemic instability, inflammation, gut–brain axis disruption, and the reward-related effects of highly palatable combinations of refined carbohydrates, fats, salt, and additives. This is clinically relevant because addictive-like eating patterns are increasingly reported: recent estimates suggest that ultra-processed food addiction affects approximately 14% of adults and 12% of children, with higher prevalence in some clinical populations [38,39]. Mental health and nutrition services should therefore be better integrated. Screening for poor diet quality, food insecurity, binge eating, and addictive-like UPF consumption should be included in primary care, obesity care, and mental health settings, particularly for patients with depression, anxiety, eating disorders, or stress-related overeating. However, nutrition care in mental health services remains poorly defined; non-nutrition clinicians often provide dietary advice without formal training, while dietitians and nutrition professionals may be underused in multidisciplinary care [40].

Clear referral pathways, trained nutrition professionals, and access to affordable minimally processed foods are therefore needed. In LMICs, community-based approaches combining nutrition education, food assistance, home gardening, maternal mental health support, and child development services may offer a practical way to reduce the cycle of psychological distress, food insecurity, and reliance on UPFs.

## 6. Conclusions

UPFs are not an inevitable feature of modern diets; they are produced and sustained by political and economic choices that shape food production, marketing, trade, pricing, and access. Addressing UPFs therefore requires more than individual nutrition advice. It requires structural action across food systems, from local procurement and food assistance programmes to marketing regulation, fiscal policy, and global trade governance. Civil society will be essential in this process by supporting policy learning, strengthening cross-sector alliances, promoting accountability, advancing food sovereignty, and advocating for financing mechanisms that prioritize health, equity, and sustainability. A life-course perspective is particularly urgent. Infant and toddler diets in many LMICs are changing rapidly, with increasing exposure to UPFs during early childhood, while child overweight and obesity are rising faster than stunting is declining. This shift may shape long-term dietary preferences and increase future risk of non-communicable diseases. Yet many existing healthy food policies, including UPF-related taxation and front-of-package warning labels, have largely overlooked infants and young children. Stronger policy action is needed to close this gap, including consideration of extending protections under the WHO International Code of Marketing of Breast-milk Substitutes to commercially produced infant and toddler foods and beverages, alongside age-appropriate warning labels and tighter restrictions on marketing to caregivers and young children.

Future research should strengthen the evidence base while avoiding overstatement of causality. Most studies linking UPF consumption with mental health outcomes remain cross-sectional, limiting causal interpretation. Longitudinal cohort studies with repeated measures of diet, psychosocial stressors, mental health, and, where feasible, neurodevelopmental or neuroimaging outcomes are needed to clarify temporal relationships and identify sensitive windows for intervention. In LMICs, surveillance systems should be expanded to monitor UPF consumption across all age groups, particularly infants and young children, while tracking the double burden of malnutrition. Key priorities include harmonizing UPF classification systems, validating rapid dietary assessment tools for low-literacy settings, and using implementation science to evaluate the real-world effectiveness, equity impacts, and feasibility of policy interventions.

Overall, UPFs are not merely a matter of dietary choice; they are a structural driver of nutritional and health inequalities. The evidence reviewed in this perspective suggests that UPF consumption intersects with food insecurity, psychosocial vulnerability, and early-life developmental susceptibility in ways that may reinforce disadvantage across the life-course. Without coordinated action, the global nutrition transition risks deepening inequalities and placing the greatest burden on the poorest and youngest populations. Many of the necessary policy tools already exist; what is needed now is the political commitment to implement them as part of an integrated, life-course strategy that places nutritional equity at the center of public health.

## Data Availability

No new data were created or analyzed in this study. Data sharing is not applicable to this article.

## References

[1] Leung C.W., Fulay A.P., Parnarouskis L., Martinez-Steele E., Gearhardt A.N., Wolfson J.A. (2022). Food insecurity and ultra-processed food consumption: The modifying role of participation in the Supplemental Nutrition Assistance Program (SNAP). Am. J. Clin. Nutr..

[2] Baraldi L.G., Steele E.M., Canella D.S., Monteiro C.A. (2018). Consumption of ultra-processed foods and associated sociodemographic factors in the USA between 2007 and 2012: Evidence from a nationally representative cross-sectional study. BMJ Open.

[3] Popkin B.M., Laar A. (2025). Nutrition transition’s latest stage: Are ultra-processed food increases in low- and middle-income countries dooming our preschoolers’ diets and future health?. Pediatr. Obes..

[4] Monteiro C.A., Cannon G., Moubarac J.C., Levy R.B., Louzada M.L.C., Jaime P.C. (2018). The UN Decade of Nutrition, the NOVA food classification and the trouble with ultra-processing. Public Health Nutr..

[5] Mottis G., Kandasamey P., Peleg-Raibstein D. (2025). The consequences of ultra-processed foods on brain development during prenatal, adolescent and adult stages. Front. Public Health.

[6] Contreras-Rodriguez O., Solanas M., Escorihuela R.M. (2022). Dissecting ultra-processed foods and drinks: Do they have a potential to impact the brain?. Rev. Endocr. Metab. Disord..

[7] Çelik Ö.M., Güler Ü., Ekici E.M. (2025). Factors Affecting Ultra-Processed Food Consumption: Hedonic Hunger, Food Addiction, and Mood. Food Sci. Nutr..

[8] Nguyen T.A., Nguyen N.N., Nguyen H.S., Linh T.T.D., Nguyen N.T.K., Hoang Y.N., Ho D.K.N. (2026). Ultraprocessed food consumption and the global burden of noncommunicable disease: A 45-country comparative risk assessment. Am. J. Clin. Nutr..

[9] Oyeneye H.O., Alghamdi A.A., Staromiejska W., Downs S.M. (2025). The Relationship Among Food Environments, Food Choices, and Nutritional Outcomes in Women and Children From Low- and Lower- Middle- Income Countries: A Systematic Review. Curr. Dev. Nutr..

[10] Bhagtani D., Adams J., Imamura F., Lahiri A., Irfan K., Jha V., Kasturiratne A., Katulanda P.J., Mridha M., Anjana R.M. (2024). OP59 Ultra-processed food consumption in South Asia: Quantification of regional variation in intakes and the assessment of their sociodemographic correlates. Findings from the South Asia Biobank in four South Asian countries. J. Epidemiol. Community Health.

[11] Kwankye S.O., Frempong-Ainguah F., Bawah A.A., Kwankye S.O., Kyei P.S. (2025). Population Dynamics in Ghana. Ghana’s Demography: Evolution and Implications for Development.

[12] World Health Organization (2012). Guideline: Sodium Intake for Adults and Children.

[13] Monteiro C.A., Cannon G., Levy R.B., Moubarac J.-C., Louzada M.L.C., Rauber F., Khandpur N., Cediel G., Neri D., Martinez-Steele E. (2019). Ultra-processed foods: What they are and how to identify them. Public Health Nutr..

[14] Petrus R.R., Sobral P.J.D.A., Tadini C.C., Gonçalves C.B. (2021). The NOVA classification system: A critical perspective in food science. Trends Food Sci. Technol..

[15] Sinha R., Jastreboff A.M. (2013). Stress as a common risk factor for obesity and addiction. Biol. Psychiatry.

[16] Dai S., Wellens J., Yang N., Li D., Wang J., Wang L., Yuan S., He Y., Song P., Munger R. (2024). Ultra-processed foods and human health: An umbrella review and updated meta-analyses of observational evidence. Clin. Nutr..

[17] Lane M.M., Gamage E., Du S., Ashtree D.N., McGuinness A.J., Gauci S., Baker P., Lawrence M., Rebholz C.M., Srour B. (2024). Ultra-processed food exposure and adverse health outcomes: Umbrella review of epidemiological meta-analyses. BMJ.

[18] Hall K.D., Ayuketah A., Brychta R., Cai H., Cassimatis T., Chen K.Y., Chung S.T., Costa E., Courville A., Darcey V. (2019). Ultra-Processed Diets Cause Excess Calorie Intake and Weight Gain: An Inpatient Randomized Controlled Trial of Ad Libitum Food Intake. Cell Metab..

[19] Di Gesù C.M., Matz L.M., Buffington S.A. (2021). Diet-induced dysbiosis of the maternal gut microbiome in early life programming of neurodevelopmental disorders. Neurosci. Res..

[20] Georgieff M.K. (2007). Nutrition and the developing brain: Nutrient priorities and measurement. Am. J. Clin. Nutr..

[21] Ferreira R.C., Marin A.H., Vitolo M.R., Campagnolo P.D.B. (2024). Early ultra-processed foods consumption and hyperactivity/inattention in adolescence. Rev. Saude Publica.

[22] DiFeliceantonio A.G., Coppin G., Rigoux L., Thanarajah S.E., Dagher A., Tittgemeyer M., Small D.M. (2018). Supra-Additive Effects of Combining Fat and Carbohydrate on Food Reward. Cell Metab..

[23] Jacka F.N., Cherbuin N., Anstey K.J., Sachdev P., Butterworth P. (2015). Western diet is associated with a smaller hippocampus: A longitudinal investigation. BMC Med..

[24] Henney A.E., Gillespie C.S., Alam U., Hydes T.J., Mackay C.E., Cuthbertson D.J. (2024). High intake of ultra-processed food is associated with dementia in adults: A systematic review and meta-analysis of observational studies. J. Neurol..

[25] García-Cabrerizo R., Carbia C., Riordan K.J.O., Schellekens H., Cryan J.F. (2021). Microbiota-gut-brain axis as a regulator of reward processes. J. Neurochem..

[26] Dunford E.K., Popkin B.M. (2023). Ultra-processed food for infants and toddlers; dynamics of supply and demand. Bull. World Health Organ..

[27] O’Connor L.E., Martinez-Steele E., Wang L., Zhang F.F., Herrick K.A. (2023). Food Processing, According to the Nova Classification System, and Dietary Intake of US Infants and Toddlers. J. Nutr..

[28] (2015). WHO Guidelines Approved by the Guidelines Review Committee. Guideline: Sugars Intake for Adults and Children.

[29] World Health Organization (2022). Nutrient and Promotion Profile Model: Supporting Appropriate Promotion of Food Products for Infants and Young Children 6–36 Months in the WHO European Region.

[30] Scrinis G., Popkin B.M., Corvalan C., Duran A.C., Nestle M., Lawrence M., Baker P., Monteiro C.A., Millett C., Moubarac J.-C. (2025). Policies to halt and reverse the rise in ultra-processed food production, marketing, and consumption. Lancet.

[31] Alderman H. (2015). Leveraging Social Protection Programs for Improved Nutrition: Summary of Evidence Prepared for The Global Forum on Nutrition-Sensitive Social Protection Programs, 2015. https://ssrn.com/abstract=2831575.

[32] Lentz E.C., Barrett C.B. (2013). The economics and nutritional impacts of food assistance policies and programs. Food Policy.

[33] Baker P., Machado P., Santos T., Sievert K., Backholer K., Hadjikakou M., Russell C., Huse O., Bell C., Scrinis G. (2020). Ultra-processed foods and the nutrition transition: Global, regional and national trends, food systems transformations and political economy drivers. Obes. Rev..

[34] World Health Organization (2021). Action Framework for Developing and Implementing Public Food Procurement and Service Policies for a Healthy Diet.

[35] Popkin B.M., Barquera S., Corvalan C., Hofman K.J., Monteiro C., Ng S.W., Swart E.C., Taillie L.S. (2021). Towards unified and impactful policies to reduce ultra-processed food consumption and promote healthier eating. Lancet Diabetes Endocrinol..

[36] Laar A., Amoah J.M., Massawudu L.M., A Pereko K.K., Yeboah-Nkrumah A., Amevinya G.S., Nanema S., Odame E.A., Agyekum P.A., Mpereh M. (2023). Making food-related health taxes palatable in sub-Saharan Africa: Lessons from Ghana. BMJ Glob. Health.

[37] Touvier M., da Costa Louzada M.L., Mozaffarian D., Baker P., Juul F., Srour B. (2023). Ultra-processed foods and cardiometabolic health: Public health policies to reduce consumption cannot wait. BMJ.

[38] Wiss D.A., LaFata E.M. (2024). Ultra-Processed Foods and Mental Health: Where Do Eating Disorders Fit into the Puzzle?. Nutrients.

[39] Poon E., Li C., Schweitzer D., Akefe I. (2025). Neurobiological insights into the effects of ultra-processed food on lipid metabolism and associated mental health conditions: A scoping review. Front. Nutr..

[40] Hiltensperger R., Neher J., Beloviene M., Bisle E., Dalton K., Gellner A.-K., Kersting X., Kolassa I.-T., Longhitano C., Lukas U. (2026). The role of nutrition professionals working in mental health settings—Insights from a multi-professional and international world café. Nutr. Psychiatry.

